# Improving Nigerian health policymakers’ capacity to access and utilize policy relevant evidence: outcome of information and communication technology training workshop

**DOI:** 10.11604/pamj.2015.21.212.6375

**Published:** 2015-07-23

**Authors:** Chigozie Jesse Uneke, Abel Ebeh Ezeoha, Henry Uro-Chukwu, Chinonyelum Thecla Ezeonu, Ogbonnaya Ogbu, Friday Onwe, Chima Edoga

**Affiliations:** 1 Department of Medical Microbiology/Parasitology, Faculty of Clinical Medicine, Ebonyi State University Abakaliki, Nigeria; 2 Health Policy & systems Research Project (Knowledge Translation Platform), Ebonyi State University Abakaliki, Nigeria; 3 Department of Banking & Finance, Ebonyi State University Abakaliki, Nigeria; 4 National Obstetrics Fistula Centre, Abakaliki, Nigeria; 5 Department of Paediatrics, Ebonyi State University Abakaliki, Nigeria; 6 Department of Applied Microbiology, Ebonyi State University Abakaliki, Nigeria; 7 Department of Sociology/Anthropology, Ebonyi State University Abakaliki, Nigeria; 8 Catholic Relief Services (Nigeria Program), Abakaliki, Nigeria

**Keywords:** Information and communication technology, policymaker, evidence-informed, capacity, workshop

## Abstract

Information and communication technology (ICT) tools are known to facilitate communication and processing of information and sharing of knowledge by electronic means. In Nigeria, the lack of adequate capacity on the use of ICT by health sector policymakers constitutes a major impediment to the uptake of research evidence into the policymaking process. The objective of this study was to improve the knowledge and capacity of policymakers to access and utilize policy relevant evidence. A modified “before and after” intervention study design was used in which outcomes were measured on the target participants both before the intervention is implemented and after. A 4-point likert scale according to the degree of adequacy; 1 = grossly inadequate, 4 = very adequate was employed. This study was conducted in Ebonyi State, south-eastern Nigeria and the participants were career health policy makers. A two-day intensive ICT training workshop was organized for policymakers who had 52 participants in attendance. Topics covered included: (i). intersectoral partnership/collaboration; (ii). Engaging ICT in evidence-informed policy making; use of ICT for evidence synthesis; (iv) capacity development on the use of computer, internet and other ICT. The pre-workshop mean of knowledge and capacity for use of ICT ranged from 2.19-3.05, while the post-workshop mean ranged from 2.67-3.67 on 4-point scale. The percentage increase in mean of knowledge and capacity at the end of the workshop ranged from 8.3%-39.1%. Findings of this study suggest that policymakers’ ICT competence relevant to evidence-informed policymaking can be enhanced through training workshop.

## Introduction

All over the world, there is increasing recognition of importance of making health policies with best available evidence. The process which is known as evidence-informed health policymaking is characterized by the systematic and transparent access to, and appraisal of, evidence as an input into policymaking [1]. According to Bowen and Zwi [2], evidence encompasses research, opinion and views of individuals or groups, results of consultative processes and published reports and documents; facts which may be actual or asserted which may be known through experience or observation. However, numerous reports have consistently indicated that evidence from research is among the most reliable category of evidence and can greatly enhance health policy development [3–6]. One of the most challenging aspects of evidence-to-policy link especially in developing countries is the capacity constraints of policymakers to access, synthesize, adapt and utilize available research evidence [7, 8]. Studies have shown that one of the major areas of policymakers’ capacity constraints in developing world is in the use information and communication technology (ICT) [9]. ICTs have been defined as “tools that facilitate communication and the processing and transmission of information and the sharing of knowledge by electronic means encompassing the full range of electronic digital and analogue ICTs” (9). ICTs have been reported to have greatly improved access to health information, research, literature and training materials, thereby supporting the health research enterprise and enabling comprehensive, evidence-based policymaking [10]. In Nigeria, the lack of adequate knowledge and capacity on the use of ICT by health sector policymakers constitute a major impediment to the uptake of research evidence into the policymaking process [11–13]. Gething and colleagues [14], had observed in their report that in most African setting, there is a paucity of ICT-related knowledge and skills limiting capacities of national health management information systems (HMIS) to generate, analyze and disseminate information for use in decision-making. The United Nations Economic commission for Africa (UNECA) recognized the importance of ICT in the improvement of the health sector in the African region. According to the UNECA report, ICTs have a crucial role to play in improving the effectiveness of the health sector by maximizing the use of scarce knowledge and limited resources as well as bringing life-enhancing knowledge to people in ways they can use, when and where they need it [15]. A number of previous published works have suggested that a relationship exists between effective job performance of a health sector stakeholder (policymaker or service provider) and ICT use [9, 10, 15]. Therefore to address the competency problem in ICT use by health sector policymakers, there is a dire need for a capacity enhancement training activity for policymakers. This is supported by Peizer [16] who in a paper on strategies to bridge the digital divide, made a case for a significant time and resource commitment to invest in training to enhance ICT competence of those involved in making health policy.

## Workshop report

### Aim of the Workshop

The aim of this study is to improve the knowledge and capacity of Nigerian policymakers to access and utilize policy relevant evidence via an ICT training workshop. In this workshop, a modified “before and after” intervention study design was used in which outcomes were measured on the eligible population (target participants) both **before** the programme (intervention) is implemented and **after** [17]. The difference between the before and after measurements was taken to be the impact of the intervention. (In this instance, the “before”- or “baseline”- measurements served as the control measurements).

### Study area and participants

This research was a sub-national study and participants consisted of individuals whose geographical area of operation is south-eastern Nigeria, with emphasis on Ebonyi State. The target participants were the career health policy makers, this category of policymakers included: health professionals in charge of the health systems; regional, state and local government directors of the health ministry; directors of primary health care at the local government level; health professionals working with specific programmes in the health ministry; staff and consultants involved in public health issues within the health ministry; programme/project managers under the health ministry; chief executive officers of civil society groups, including non-governmental organizations; leaders of national health-based associations (for example, Nigerian Medical Association; National Association of Nigeria Nurses and Midwives; and Pharmaceutical Association of Nigeria) This category of policymakers were selected as key participants in this study because findings from our previous studies [7, 18, 19] clearly indicate that at the State and Local Government levels, they play the most vital role in “evidence-to-policy” making process. They are the principal actors involved in the generation, collection and assembling of policy relevant information, and processing of data and reports on health-related issues from the different sectors of the health system. They also prepare these into forms that can be submitted for the drafting of policy documents.

### Ethical consideration

Approval for this study was obtained from the Directorate of Research, Innovation & Commercialization (DRIC), Ebonyi State University, Abakaliki Nigeria. The approval was based on the agreement that participation in the research was voluntary following informed consent; that participants’ anonymity would be maintained; and that every finding would be treated with utmost confidentiality and for the purpose of this research. These were adhered to in this study.

### Facilitators

A total of five facilitators were engaged in the workshop based on their expertise in the training contents. The facilitators included four senior academics from Ebonyi State University Abakaliki Nigeria and a senior director from the Ministry of Health. The academics included a Professor of infectious diseases and epidemiology with vast experience in intersectoral partnership/ collaboration in health policymaking; the remaining three senior academics have vast experience in evidence-informed policy making and knowledge translation in the Nigeria context. The only policymaker facilitator has vast experience working with the government in the formulation of policy in the south eastern Nigeria.

### Pre-Workshop tasks

A total of 70 policymakers were mapped out for this study. The mapped out participants were divided into two batches of 35 persons each. We organized a two-day intensive health-policy based ICT training workshop at the Ebonyi State University, Information and Communication Technology (ICT) (E-Library) Centre Abakaliki Nigeria, for the policymakers. All the policymakers were invited to the workshop by invitation letters which were sent 2 weeks before the event and was followed-up with a text message reminder to their mobile phones a day before the programme.

### Programme

The first batch of policymakers had their workshop in April 2014, while the second batch had theirs in May 2014. The duration of the workshop each day was five hours from 10am-3pm (with a break between 12:30pm-1pm). A pre-workshop assessment questionnaire (developed in a 4-point likert scale according to the degree of adequacy; 1 = grossly inadequate, 4 = very adequate), was administered prior to actual training to assess the level of knowledge and capacity of the participants on the specific topics to be covered within the theme of the workshop. After the administration of the pre-workshop questionnaire the training commenced and was facilitated by four resource persons (three senior researchers from Ebonyi State University and one senior director from the health ministry). The workshop covered the following topics: (i). Need for intersectoral partnership/ collaboration in health policymaking in Ebonyi State; (ii). Benefits of engaging ICT in evidence-informed policy making for control of infectious diseases of poverty and running the health sector; (iii). Introduction to use of ICT for evidence synthesis;(iv) Development of the capacity of the policymakers on the use of computer, internet and other ICT the following: (a). The search protocol for health information and policy relevant research evidence; (b). Identification of and search strategies for evidence of a wide range of electronic resources, in addition to the traditional scientific and clinical databases (eg., MEDLINE, EMBASE, Cochrane Database, Allied and Complementary Medicine, British Nursing Index, Social Policy and Practice etc); (c). Information/evidence audit to accompany search strategies.

All teaching sessions were done using power-point presentation and handouts on each topic were produced and distributed to all participants. It was made mandatory for all lectures to be delivered in simplified, practical and easily comprehensible patterns, with little or no emphasis on complex mathematical or scientific computations/models for the benefit of non-specialists who constituted the majority of the participants. Practical sessions were held during the workshop in which each participant was able to use an internet connected computer to practice the acquisition of research evidence from relevant electronic databases. At the end of the workshop, a post-workshop assessment questionnaire was administered to the participants to evaluate the impact of the workshop.

### Evaluation

The data collected via the questionnaire (developed in a likert scale format) was analyzed using the methods developed at McMaster University Canada by Johnson and Lavis [20]. The analysis is based on mean rating (MNR), median rating (MDR) and range. For instance the figures represent Likert rating scale of 1-4 points, where 1 point=grossly inadequate; 2 points=inadequate; 3 points=fairly adequate; and 4 points=very adequate. In terms of analysis, values ranging from 1.00-2.49 points are considered low, whereas values ranging from 2.50-4.00 points considered high. The Pre-ICT means was compared to the Post-ICT means. The EPi-info software was used for the performance of the data analysis.

### Outcomes

Out of the 70 policymakers and stakeholders invited for the workshop, a total of 52 individuals attended. The official designation attributes as indicated in Table 1 included the following: Programme Officer/Project Secretaries (28.8%); Managers/Heads of Departments (30.8%); Directors/Presidents/Chairpersons (40.4%). A total of 34.6% of the participants have direct influence on the policymaking process, with 51.9% and 30.8% possessing Bachelors and Masters Degrees as highest academic qualifications respectively. The outcome of PRE-ICT knowledge and skill assessment is presented in Table 2, while the POST-ICT knowledge and skill assessment is presented in Table 3. The outcome of the assessment of the impact of the training workshop with the comparison of the PRE-ICT mean and POST-ICT mean is presented in Table 4.

**Table 1:** Attributes of the participants during the ICT Training for the Research Capacity Strengthening and Knowledge Management for policymakers in Ebonyi State Nigeria

Participant (Respondents) Attributes	Number (%) of Participants (Respondents) N=52
**(1) Gender**	
Female	20 (38.5)
Male	31 (59.6)
(Missing)	1 (1.9)
**(2) Age (Years)**	
25 - 34	3 (6.1)
35 - 44	14 (28.6)
≥ 45	32 (65.3)
**(3) Institutional Affiliation**	
Federal Teaching Hospital	12 (23.1)
State Ministry of Health	16 (30.8)
Local Government Service Commission	20 (38.5)
Non-Governmental Organization	3 (5.8)
State House of Assembly	1 (1.9)
**(4) Official Designation**	
Programme Officer/Project Secretaries	15 (28.8)
Managers/Heads of Departments	16 (30.8)
Directors/Presidents/Chairpersons	21 (40.4)
**(5) Years of Experience in Current Designation**	
< 3	9 (18.4)
3 – 5	20 (40.8)
6 – 10	17 (34.7)
> 10	3 (6.1)
(Missing)	3 (6.1)
**(6) Influence on Policy Making**	
Direct (DIPP)	18 (34.6)
Indirect (IIPP)	34 (65.4)
**(7) Highest Academic Qualification**	
SSCE/Diploma	5 (9.6)
Bachelor	27 (51.9)
Masters	16 (30.8)
Doctorate	4 (7.7)

**Table 2:** The Pre-ICT knowledge/skill assessment of participants at the ICT Training for the Research Capacity Strengthening and Knowledge Management for policymakers in Ebonyi State Nigeria

Parameter Assessed	Mean	Median	Mode	Range
Knowledge about the Importance and characteristics of inter-sectoral partnership/collaboration in health policy making	3.00	3.00	4	1-4
Knowledge about the different sectors that can collaborate in health policy making	3.05	3.00	3	1-4
Ability to identify the different requirements for inter-sectoral partnership/collaboration in health policy making	2.84	3.00	3	1-4
Knowledge about Factors that hamper inter-sectoral partnership/collaboration in health policy making	3.00	3.00	3	2-4
Knowledge about factors that can enhance inter-sectoral partnership/collaboration in health policy making	2.97	3.00	3	1-4
Knowledge of Use of ICT for running the health sector and infectious disease control	2.35	2.00	2	1-4
Knowledge about the use of ICT in improving the functioning of health care systems	2.55	2.50	2	1-4
Knowledge about the use of ICT for improving the delivery of health care	2.35	2.00	3	1-4
Knowledge about the use of ICT for improving communication about health	2.51	3.00	3	1-4
Knowledge about the constraints and challenges of use of ICT in health sector	2.55	3.00	3	1-4
Knowledge about how to address challenged and improve the use of ICT in the health sector	2.26	2.00	2	1-4
Knowledge on the importance and benefits of the internet	3.03	3.00	3	1-4
Ability to create and use email address	2.66	3.00	2	1-4
Ability to locate information on the internet	2.64	3.00	2	1-4
Ability to locate and access websites of different organizations	2.32	2.00	2	1-4
Knowledge on the types and use of major search engines	2.24	2.00	3	1-4
Ability to locate and access relevant databases	2.21	2.00	2	1-4
Knowledge about the use of ICT for accessing information for policy making	2.32	2.00	2	1-4
Knowledge about what research evidence is and the different types used in policy making	2.49	3.00	3	1-4
Knowledge about the sources of evidence and electronic databases relevant for evidence in policy making	2.19	2.00	2	1-4
Ability to search and identify existing research evidence in policy making context	2.32	2.00	2	1-3
Ability to access and use existing research evidence in policy making	2.38	2.00	3	1-3
Capacity to assess the authenticity, validity, reliability, high quality of research evidence	2.29	2.00	2	1-4
Capacity to assess the relevance and applicability of research evidence	2.32	2.00	2	1-3

**Table 3:** The POST-ICT knowledge/skill assessment of participants at the ICT Training for the Research Capacity Strengthening and Knowledge Management for policymakers in Ebonyi State Nigeria

Parameter Assessed	Mean	Median	Mode	Range
Adequacy of Training in relation to Job Description	3.57	4.00	4	2-4
Rating of the quality of the training	3.58	4.00	4	2-4
Rating of the impact of the training	3.66	4.00	4	2-4
Present knowledge about the Importance and characteristics of inter-sectoral partnership/collaboration in health policy making	3.67	4.00	4	3 -4
Present knowledge about the different Sectors that can collaborate in health policy making	3.49	3.00	3	3-4
Present ability to identify the different requirements for inter-sectoral partnership/collaboration in health policy making	3.37	3.00	3	2-4
Present knowledge about Factors that hamper inter-sectoral partnership/collaboration in health policy making	3.58	4.00	4	2-4
Present knowledge about factors that can enhance inter-sectoral partnership/collaboration in health policy making	3.64	4.00	4	1-4
Present knowledge of Use of ICT for running the health sector and infectious disease control	3.16	3.00	3	2-4
Present knowledge about the use of ICT in improving the functioning of health care s	3.19	3.00	3	1-4
Present knowledge about the use of ICT for improving the delivery of health care	3.27	3.00	3^a^	1-4
Present knowledge about the use of ICT for improving communication about health	3.41	3.00	4	2-4
Present knowledge about the constraints and challenges of use of ICT in health sector	3.27	3.00	3	2-4
Present Knowledge about how to address challenged and improve the use of ICT in the health sector	3.14	3.00	3	1-4
Present knowledge on the importance and benefits of the internet	3.51	4.00	4	2-4
Present ability to create and use email address	3.06	3.00	3	1-4
Present ability to locate information on the internet	2.86	3.00	3	1-4
Present ability to locate and access websites of different organizations	2.81	3.00	3	1-4
Present knowledge on the types and use of major search engines	2.78	3.00	3	1-4
Present ability to locate and access relevant databases	2.69	3.00	3	1-4
Present knowledge about the use of ICT for accessing information for policy making	3.08	3.00	3	2-4
Present Knowledge about what research evidence is and the different types used in policy making	3.03	3.00	3	2-4
Present knowledge about the sources of evidence and electronic databases relevant for evidence in policy making	2.70	3.00	3	1-4
Present ability to search and identify existing research evidence in policy making context	2.97	3.00	3	2-4
Present ability to access and use existing research evidence in policy making	2.78	3.00	3	1-4
Present capacity to assess the authenticity, validity, reliability, high quality of research evidence	2.67	3.00	3	1-4
Present capacity to assess the relevance and applicability of research evidence	2.91	3.00	3	2-4

**Table 4:** Comparison of the Pre-ICT and Post-ICT knowledge/skill assessment of participants at the ICT Training for the Research Capacity Strengthening and Knowledge Management for policymakers in Ebonyi State Nigeria

Parameter Assessed	PRE+Mean	POST++Mean	Mean Increase	Percentage Mean Increase
***INTERSECTORAL PARTNERSHIP/ COLLABORATION IN HEALTH POLICYMAKING IN EBONYI STATE***				
Knowledge about the Importance and characteristics of inter-sectoral partnership/collaboration in health policy making	3.00	3.67	0.67	22.3%
Knowledge about the different sectors that can collaborate in health policy making	3.05	3.49	0.44	14.3%
Ability to identify the different requirements for inter-sectoral partnership/collaboration in health policy making	2.84	3.37	0.53	18.7%
Knowledge about Factors that hamper inter-sectoral partnership/collaboration in health policy making	3.00	3.58	0.58	19.3%
Knowledge about factors that can enhance inter-sectoral partnership/collaboration in health policy making	2.97	3.64	0.67	22.6%
***ENGAGING ICT IN EVIDENCE-INFORMED POLICY MAKING FOR CONTROL OF INFECTIOUS DISEASES OF POVERTY AND IN RUNNING THE HEALTH SECTOR***				
Knowledge of Use of ICT for running the health sector and infectious disease control	2.35	3.16	0.81	34.5%
Knowledge about the use of ICT in improving the functioning of health care systems	2.55	3.19	0.64	25.1%
Knowledge about the use of ICT for improving the delivery of health care	2.35	3.27	0.92	39.1%
Knowledge about the use of ICT for improving communication about health	2.51	3.41	0.90	35.9%
Knowledge about the constraints and challenges of use of ICT in health sector	2.55	3.27	0.72	28.2%
4vi. Knowledge about how to address challenged and improve the use of ICT in the health sector	2.26	3.14	0.88	38.9%
***KNOWLEDGE & APPLICATION OF INFORMATION/COMMUNICATION TECHNOLOGY***				
5Ai. Knowledge on the importance and benefits of the internet	3.03	3.51	0.48	15.8%
Ability to create and use email address	2.66	3.06	0.40	15.0%
Ability to locate information on the internet	2.64	2.86	0.22	8.3%
Ability to locate and access websites of different organizations	2.32	2.81	0.49	21.1%
Knowledge on the types and use of major search engines	2.24	2.78	0.54	24.1%
Ability to locate and access relevant databases	2.21	2.69	0.48	21.7%
***USE OF ICT FOR EVIDENCE SYNTHESIS***				
Knowledge about the use of ICT for accessing information for policy making	2.32	3.08	0.76	32.8%
Knowledge about what research evidence is and the different types used in policy making	2.49	3.03	0.54	21.7%
Knowledge about the sources of evidence and electronic databases relevant for evidence in policy making	2.19	2.70	0.51	23.3%
Ability to search and identify existing research evidence in policy making context	2.32	2.97	0.65	28.0%
Ability to access and use existing research evidence in policy making	2.38	2.78	0.40	16.8%
Capacity to assess the authenticity, validity, reliability, high quality of research evidence	2.29	2.67	0.38	16.6%
Capacity to assess the relevance and applicability of research evidence	2.32	2.91	0.59	25.4%

+ PRE-ICT Training Workshop Mean;

++ POST- ICT Training Mean

Result showed a progressive increase in the POST-ICT mean over the PRE-ICT mean. In terms of the “*intersectoral partnership/ collaboration in health policymaking*”, the PRE-ICT mean ranged from 2.99-3.05, while the POST-ICT mean ranged from 3.37-3.67, with the percentage increase ranging from 14.3%-22.6%. In terms of “*engaging ICT in evidence-informed policy making for control of infectious diseases of poverty and in running the health*”, the PRE-ICT mean ranged from 2.26-2.55, while the POST-ICT mean ranged from 3.14-3.41, with the percentage increase ranging from 25.1%-39.1%. In terms of “*knowledge & application of information/communication technology for Internet use*”, the PRE-ICT mean ranged from 2.21-3.03, while the POST-ICT mean ranged from 2.69-3.51, with the percentage increase ranging from 8.3%-24.1%. Concerning the “*use of ICT for evidence synthesis*”, the PRE-ICT mean ranged from 2.19-2.49, while the POST-ICT mean ranged from 2.67-3.08, with the percentage increase ranging from 16.6%-32.8% (Table 4).

## Discussion

The result of this study clearly showed a notable improvement in the knowledge and capacity of the participants regarding the use of ICT to access, synthesize and apply relevant evidence in policymaking. This notable improvement is demonstrated by the tremendous increase in the POST-ICT mean of knowledge/skill over that of the PRE-ICT mean in all the subject areas covered at the training workshop. This outcome suggests that the training workshop strategy we employed in the study, appeared to produce the desired capacity enhancement among the participating policymakers. Evidence that emerged from this study suggests that an ICT training workshop can serve as an intervention mechanism towards improving the ability of policymakers and other stakeholders to use ICT. We observed a similar trend in our previous study [13]. The report of HIFA [21] highlighted the many strategic benefits of training workshops (when used as in-service training) to include: presenting new information to groups of people, practicing new skills and allowing health workers to share experiences and insights. Furthermore HIFA [21] noted that teaching delivered to groups is often thought to be economical and another advantage is that it is thought that adults learn best by sharing of experience, discussion and doing. Gates [22] had earlier indicated that using training workshop a significant impact on skill difference was gained on providers’ skills in assessing and managing HIV/AIDS patients. Methods used in the workshops such as the administration of pre-workshop questionnaire and post-workshop questionnaire; general discussions; group works and short presentations have been shown to be very effective in capacity enhancement [23]. The ICT capacity enhancement training workshop was conducted as part of the strategies designed to improve the ability of the policymakers to be able to acquire and assess policy relevant information. The result of our study indicated that “*Knowledge about the use of ICT for accessing information for policy making” and “Knowledge about how to address challenges and improve the use of ICT in the health sector*” were among the capacity issues that recorded the highest percentage improvement (32.8% and 38.9% respectively) (Table 4). We focused on the improvement of the capacity and skill to use ICT in the health policymaking process because ICTs have been noted to have the potential to make a major contribution to improving access and quality of services while containing costs [9], and could provide fast, efficient and relatively cheap access to information leading to dramatic improvements in access to advice and care [10]. Reports by UNESCO [24] and WHO [25] noted that we are living in what are increasingly referred to as “knowledge societies” which are able to harness the huge amount of information that modern technology such as computers and the Internet allow us to manipulate, store, transmit and share. According to Green and Bennett [26], the skill, therefore, lies in turning all this information into knowledge; and the great challenge is to then use that knowledge-to put it into practice. We designed the ICT training workshop content as a comprehensive capacity enhancement mechanism based on topics that we are convinced have potential to improve the evidence-to-policy link. The topics including engaging ICT in evidence-informed policy making and in running the health sector; knowledge & application of ICT for Internet use, information literacy; defining information problem; searching for information online; and evaluating information have been described as areas requiring capacity strengthening for policy makers in low-income settings [9].

In the present study, it is worthy of note that the knowledge of the use of ICT for evidence synthesis improved considerably among the policymakers after the training workshop. The percentage increase ranged from 16.6% to as high as 32.8%. The implication of this is that the policymakers have acquired some abilities to use of ICT for accessing policy relevant information; know the sources of evidence and electronic databases relevant for evidence in policy making; and to search and identify existing research evidence in policy making context; as well as assess the authenticity, validity, reliability, high quality of research evidence. There are numerous reports including randomized controlled trials that have demonstrated considerable improvement in the capacity of health professionals to use ICT effectively to improve healthcare capacity following training/instructional programme/workshops [27–31]. It is based on this premise that a number of international agencies including the United Nations Asian and Pacific Training Centre for Information and Communication Technology for Development's (UN-APCICT) are promoting capacity enhancement on ICT among policymakers [32]. The UN-APCICT has undertaken training workshops for policymakers in a number of countries in Asia and noted that such training programmes can equip government officials with the knowledge and skills they need to fully utilize the potential of ICTs to achieve national development goals and bridge the digital divide [32–35]. Our expectation from this study therefore, is that the enhanced capacity that policymakers have acquired from the training workshop will enable them to seek, advocate for and use available research evidence in the policymaking process. In an earlier report Daly [36], had argued that with enhanced ICT capacity and given the right policies, organization, resources and institutions, ICTs can be powerful tools in the hands of those who make health policy and those working to improve health.

## Conclusion

It is pertinent to state that although notable improvement in knowledge and skill was witnessed in this study among the participants, the study was not without a number of limitations. First, the ICT training workshop lasted only two days. This could be considered rather too short a time duration to assess the real impact of the training. Furthermore the post-workshop assessment that was conducted may not have been very adequate to evaluate the impact of the training skills acquired. A follow-up of participants to see how far they are able to use the skill acquired in the policymaking process would have provided a more excellent evaluation as we observed in our previous study [13]. The findings of this study notwithstanding these limitations have demonstrated that policymakers’ ICT competence can be enhanced through training workshops as demonstrated in this study in Ebonyi State Nigeria.
